# Compound Heterozygous MYO5B Mutation, a Cause of Infantile Cholestasis: A Case Report

**DOI:** 10.31729/jnma.7860

**Published:** 2022-09-30

**Authors:** Muna Khanal, Adarsh Kumar Jha, Arun Kumar Sharma

**Affiliations:** 1 Nepal Medical College and Teaching Hospital, Jorpati, Kathmandu, Nepal; 2 Department of Paediatrics, Alka Hospital, Jawalakhel, Kathmandu, Nepal

**Keywords:** *case reports*, *intrahepatic cholestasis*, *genetic testing*

## Abstract

Infantile cholestasis is a common clinical problem in early infancy characterised by impairment in bile formation and/or flow. It requires prompt evaluation for underlying aetiology to initiate appropriate management. Although biliary atresia remains the most important aetiology, metabolic and monogenic disorders are increasingly identified with advances in diagnostic genetic testing. Progressive familial intrahepatic cholestasis disorders characterised by defects in biliary canalicular transport are among the most common monogenic disorders of cholestasis. Homozygous or compound heterozygous mutation in the Myosin 5B gene leading to a progressive familial intrahepatic cholestasis-like phenotype with or without intestinal features of microvillus inclusion disease is a relatively recently identified disorder. The incidence of these newer variants of progressive familial intrahepatic cholestasis is not yet known due to the paucity of studies. We report an uncommon cause of refractory cholestasis reported in a girl who presented with severe pruritus as the primary manifestation.

## INTRODUCTION

Neonatal/Infantile cholestasis (NIC) is a defect in bile formation and/or flow that causes liver injury whose early diagnosis can enhance outcomes.^1^ Genetic mutation accounting for 20-25% of cases of cholestasis represents the second most common group of illnesses after biliary atresia.^2^ Progressive familial intrahepatic cholestasis (PFIC) due to impaired biliary canalicular transport is a common monogenic cholestatic illness, although newer mutations with a similar phenotype have been identified. Myosin 5B (MYO5B) cholestasis is one such disorder exact incidence of which is not yet known due to the paucity of studies.^3^ This case reports a girl with MYO5B mutation presenting with a PFIC-like phenotype for the first time in Nepal.

## CASE REPORT

A 10-months-old infant born to non-consanguineously married parents at term weighing 3200 gms presented to our outpatient clinic with excessive pruritus disturbing sleep since early infancy but had increased in severity over the preceding 2 months. Her mother also described occasional episodes of yellowish-white to watery loose stools without blood or mucus that would resolve on their own. Parents had not noted any significant jaundice in the neonatal period or later infancy. There was no history to suggest other systemic illnesses. The parents were asymptomatic, and the family history was non-contributory to any jaundice or liver disorders. She was exclusively breastfed till 5 months and subsequently weaned with cereals.

On examination, she weighed 7.8 kgs and was alert and afebrile but irritable. Multiple excoriation marks were seen throughout the body that had been treated as eczema by many practitioners. She was not overtly icteric, but a doubtful yellowish tinge was noted in her sclera. No evidence of an apparent metabolic disorder was noted. Abdominal examination identified a palpable liver about 2 cm below the costal margin without splenomegaly or ascites. She seemed to be developing normally for her age. The rest of the examination was unremarkable.

Initial laboratory evaluation revealed mild eosino-philia and identified cholestasis. Her white cell count was 19,700/μl (N20 L74) with an absolute eosinophil count of 591/μl. The hemogram was otherwise within normal limits: haemoglobin 11.4 gm/dl and platelets count 496,000/cumm. The liver function test showed mildly elevated bilirubin with a notable direct fraction (bilirubin: Total 2.9 mg/dl and direct 1.3 mg/dl) with normal transaminases but markedly elevated alkaline phosphatase (974 U/l) in the setting of normal gamma-glutamyl transpeptidase (GGT) (24 U/l). The synthetic function of the liver was normal, as evidenced by normal serum albumin and prothrombin time. Her thyroid function was normal, and an extended tandem mass spectroscopy for multiple inherited metabolic disorders did not reveal any abnormality. An ultrasound of the abdomen did not show any evidence of biliary tract disease or dilatation of intrahepatic biliary radicals. She was started on ursodeoxycholic acid and supplemented with water- and fat-soluble vitamins as part of the management of cholestasis with a plan for liver biopsy.

During follow-up, she developed overt jaundice following an apparent viral illness. Her liver functions continued to show cholestasis without any transaminitis (Total: bilirubin 4.2 mg/dl, direct bilirubin 3.2 mg/dl, serum glutamic-oxaloacetic transaminase (SGOT) 57 U/l, alkaline phosphatase 1785 U/l, GGT 37 U/l). Her prothrombin time was, however, deranged to 37 seconds but was quickly corrected with parenteral vitamin K. Overt jaundice resolved within a week. A percutaneous liver biopsy was performed based on joint North American Society for Pediatric Gastroenterology, Hepatology and Nutrition/European Society for Paediatric Gastroenterology Hepatology and Nutrition (NASPGHAN/ ESPGHAN) guidelines for the evaluation of cholestatic disorders.^4^

**Figure 1:**
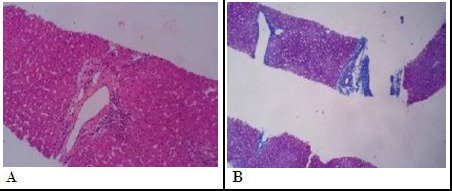
A) Mild portal chronic inflammation, B) Scattered foci of chronic lobular inflammation consisting of lymphocytes.

The liver biopsy revealed marked canalicular bile stasis. Scattered hepatocytic giant cells were seen with multiple nuclei. Bile ducts were well represented, without ductopenia. Viral cytopathic changes and steatosis were not seen. Periodic Acid Schiff-diastase (PAS/D) stain was negative for alpha 1 antitrypsin globules. Histological findings suggested cholestatic hepatitis with grade 1/4 and stage 0/4 (grading and staging as per Batts and Ludwig). The liver biopsy conclusively ruled out biliary atresia, the most common cause of infantile cholestasis, and primary sclerosing cholangitis was considered unlikely with the available evidence (Figure 1).

She was continued on ursodeoxycholic acid, antihistamines, and vitamin supplements. Her follow-up liver function tests continued to show cholestasis with normal GGT. She had occasional diarrhoea without significant dehydration and did not develop clinically apparent pancreatic insufficiency. In view of her clinical course with persistent cholestasis and one episode of overt jaundice, she was considered to represent one of the PFIC phenotypes and was subjected to genetic testing. Genome sequencing with next-generation sequencing (NGS) was performed for pathogenic variations. A heterozygous single base pair deletion in exon 19 of the MYO5B gene was identified, which resulted in a frameshift mutation, and premature truncation of protein five amino acids downstream to codon 766 was detected and considered pathogenic. Additionally, a heterozygous missense variation in exon 20 of MYO5B was also detected, resulting in cysteine substitution for arginine at codon 824. This mutation has been previously reported in patients affected with cholestasis and was considered likely pathogenic.^5^

Subsequently intestinal biopsy was performed which ruled out the possibility of microvillus inclusion disease. Based on all clinical features and laboratory evaluation, a diagnosis of MYO5B gene mutation leading to infantile cholestasis presenting with PFIC phenotype was made. She is currently followed up for progression of liver disease but continues to have refractory pruritus poorly responsive to therapy. Parents have been advised to undergo Sanger sequencing for confirmation of the mutation. They have also been notified about a future possible need for hepatic transplantation.

## DISCUSSION

This case reports an unusual cause of cholestasis due to a mutation in the MYO5B gene. Different forms of biliary atresia represent the most common cause of infantile cholestasis. Still, various other medical conditions are also known to result in refractory cholestasis, including genetic, metabolic, endocrine, hematologic, and infectious conditions. Identified genetic factors include deficiencies in canalicular bile acid transport, tight junction defects, inborn errors of bile acid metabolism, inborn errors of metabolism unrelated to bile acid production, and other syndromic/systemic disorders.^1^

The increasing availability of genetic testing with next-generation sequencing has helped to identify many genetic disorders by identification of possibly pathogenic variants which mostly follow autosomal recessive inheritance patterns, but there is growing evidence that single heterozygous pathogenic variants may also predispose to disease. These heterozygous variants' role in disease causality is complicated because of uncertainties in predicting pathogenicity, the potential presence of unidentified causal variants somewhere else in the genome, and phenotypic variability among patients. Hence, correlation of clinical, biochemical, and genetic findings is essential to implicate the identified variation in disease causality. Our patient had most of the clinical and biochemical features in addition to the observed genetic variation to help us diagnose MYO5B cholestasis.^2^

Apart from cystic fibrosis and alpha 1 antitrypsin deficiency, PFIC syndromes represent important genetic disorders of infantile cholestasis. It has been subclassified into various disease patterns based on specific bile transporter defects. The three most common types are caused by a mutation in ATP8B1, ABCB11, and ABCB4. Recent genetic advances and molecular analysis have identified three more variants to produce a similar phenotype with a mutation in NR1H4, FXR, and MYO5B genes. Although it has not yet been recognized by the Online Mendelian Inheritance in Man database, the MYO5B mutation, known to cause Microvillous Inclusion Disease (MVID), is also believed to produce isolated cholestasis and is occasionally referred to as PFIC 6.^3^

Our patient had normal-low GGT intrahepatic cholestasis and minimal bowel symptoms that closely resemble classical PFIC but was identified to have MYO5B mutation which is classically associated with microvillus inclusion disease. The age of appearance of first symptoms and clinical course of our patient was around 5 months, similar to the pattern reported in the first series of five patients with MYO5B mutations with progressive familial intrahepaticcholestasis-like phenotype with normal serum gamma-glutamyl transferase activity without intestinal disease.^6^

In another study where 114 patients were reviewed, it was demonstrated that a nearly complete genotype-phenotype correlation exists with three different phenotypes for different MYO5B genotypes: MYO5B-PFIC, pure MYO5B-MVID, and MYO5B-MIXED. MYO5B-PFIC is a phenotype with predominant cholestatic liver disease, which is clinically indistinguishable from low-GGT PFIC.^7^ This clinical phenotype is similar to our reported case with refractory cholestasis with minimal bowel involvement.

In another study, an Indian child was reported with similar episodes of severe fluctuating jaundice, pruritus, and absence of diarrhoea. Exon 19 mutation in MYO5B gene was also identified in their patient like in our patient in addition to another mutation in Exon 14. Both the asymptomatic father and mother of the patient also had heterozygous mutations in exon 14 and exon 19 of the MYO5B gene, respectively.^8^ Another study reported a 15-year-old girl similar phenotype with compound heterozygous mutations in the MYO5B gene (chr18:47462664T>C; p.Tyr654Cys and chr18:47480695G>C; p.His552Gln) who was treated surgically with cholecysto-jejuno-colonic anastomosis that significantly reduced pruritus as a potential avenue for therapy of these patients.^9^

This case highlights the importance of genetic evaluation of infants with cholestasis and its possible role in planning treatment modalities. It is possible to perform deletion/duplication analysis, coding area sequence analysis, and targeted variant analysis. For the successful molecular genetic diagnosis of neonatal/infantile intrahepatic cholestasis, targeted next-generation sequencing (NGS) has been most frequently used.^10^

Hence, target gene sequencing can help diagnose the cause of cholestasis and detect variants of mutation potentially leading to the phenotype as in this report with compound heterozygous mutation in the MYO5B gene detected for the first time in the Nepali population.
